# Supplementary figures and images for: Three new Penicillium species isolated from the tidal flats of China
Source: PeerJ. 2022 May 6;10:e13224. doi: 10.7717/peerj.13224 (PMC9083529; doi:10.7717/peerj.13224)

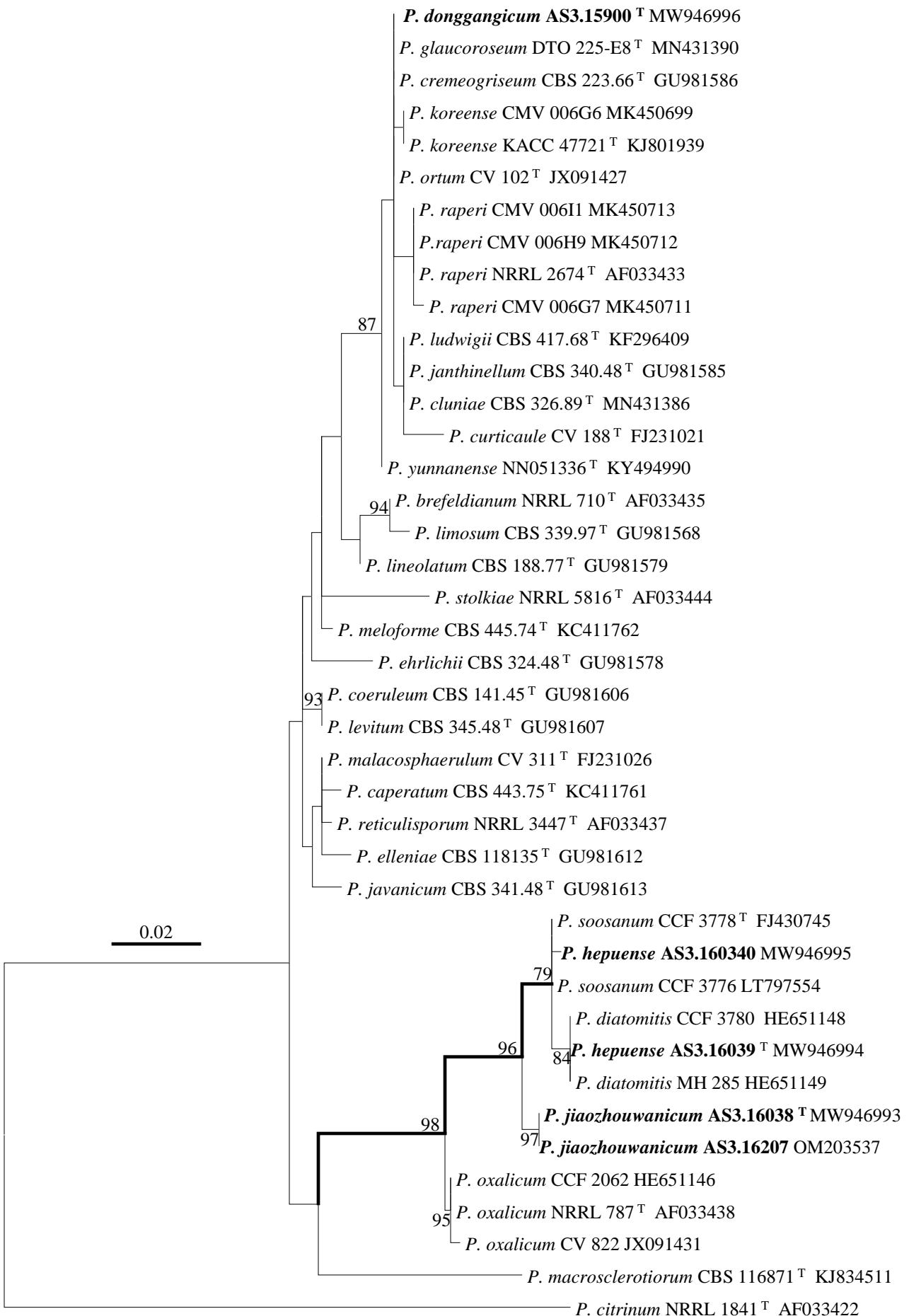

Supplement: Figure S1 — Bootstrap percentages over 70% derived from 1000 replicates are indicated at the nodes, T indicates ex-type strains, strains belonging to new species are indicated in boldface. Bar = 0.02 substitutions per nucleotide position. [file peerj-10-13224-s001.pdf]
